# Supplementary material for: Patterns of Intron Gain and Loss in Fungi
Source: PLoS Biol. 2004 Nov 30;2(12):e422. doi: 10.1371/journal.pbio.0020422 (PMC532390; doi:10.1371/journal.pbio.0020422)
Supplement: Table S1 — Also available at http://genes.mit.edu/NielsenEtAl/. (4.3 MB ZIP). [file pbio.0020422.st001.zip › NielsenEtAl/html/705.html]

AN3932.1.NCU09309.1.MG00170.1.FG06886.1


```
 CLUSTAL W (1.82) Multiple Sequence Alignments - Introns Inserted


Sequence 1: NCU09309.1	291 aa
Sequence 2: MG00170.1	284 aa
Sequence 3: FG06886.1	283 aa
Sequence 4: AN3932.1	296 aa
Alignment Length: 306 aa
Number Identitical Residues: 210 aa
Alignment Score (without introns) 8945


MG00170.1 	MDTLVARYSRPAYQQNETFTEDDQQDLCDSVP-SLSLKFAVPPVAH0PSSWLRTATDDHA
NCU09309.1	MDALVARYSRPAYQQNELFSEEEQQDLCETQP-PLSLKFAMPPVAH0PSSWLRAATDDRA
FG06886.1 	MNSLVSQYSRSSYEQNEPLDE--QDELLNSMGGDLSIKFAMPPVAQ0PSSWLRAATDDRS
AN3932.1  	MDQLVRQYTRSPH-QNEFYSEQEQHDLTESLP-PLSLKFALPPVAN0QSAFLRAMTDDHS
          	*: ** :*:*..: ***   *..*.:* ::    **:***:****:  *::**: ***::

MG00170.1 	NPNCPIKIAHGTTTLAFRFQGGI~IVATDSRATAGNWIASQTVKKVIEIN----------
NCU09309.1	NPSCPIKIAHGTTTLAFRFQGGI~IVATDSRATAGNWIASQTVKKVIEIN----------
FG06886.1 	NPNCPIKIAHGTTTLAFRFQGGI~IVATDSRATAGNWIASQTVKKVIEIN----------
AN3932.1  	NPNCPIKLAHGTTTLAFRFQGGI0IVCTDSRATAGNWIASQTVKKVIPVSRLSRGEDKPG
          	**.****:*************** **.******************** :.  : ......

MG00170.1 	----SCLLGTMAGGAA~DCQYWLAWLGMQCRLHE~LRHKRRISVAAASKILANLVYQYKG
NCU09309.1	----SDLLGTMAGGAA~DCQYWLAWLGMQCRLHE~LRHKRRISVAAASKILANLVYQYKG
FG06886.1 	----SVLLGTMAGGAA~DCQYWLAWLGMQCRLHE~LRHKRRISVAAASKILANLVYSYKG
AN3932.1  	SAPVPGLLGTMAGGAA0DCQYWLRYLSQQCTLHE1IRHKRRITVAAASKILANLTYAYKG
          	::. . ********** ****** :*. ** *** :******:***********.* ***

MG00170.1 	MGLSMGTMCAG~VTKEEGPALYYIDSDGTRLAGNLFCVGSGQTFAYGVLDAEYKYDLSDE
NCU09309.1	MGLSMGTMCAG~VTKEEGPALYYIDSDGTRLAGNLFCVGSGQTFAYGVLDAEYRYDLTVE
FG06886.1 	MGLSMGTMCAG~VTKEEGPALYYVDSDGTRLAGNLFCVGSGQTFAYGVLDAEYKYELSDE
AN3932.1  	YGLSMGTMLAG0VTPQEGPALYYIDSDGTRLPGNLFCVGSGQTFAYGVLDANYRYDLTEE
          	 ******* ** ** :*******:*******.*******************:*:*:*: *

MG00170.1 	DALELGRRSILAATHRDAYSGGFINLYHVKEDGWVKHGFNDTNPIFWKTKLEKGEFTNVT
NCU09309.1	EALELGSRSILAATHRDAYSGGFINLYHVKESGWEKHGFNDTNPIFWRTKLEKGEFSNVT
FG06886.1 	EALELGKRSILAATHRDAYSGGYINLYHVKEEGWVKHGFNDTNPIFWKTKLEKGEFTNVT
AN3932.1  	EALELGRRSILAAMHRDAYSGGFINLYHVKEEGWVHHGFDDMNPIFWKTKLEKGEFSNVT
          	:***** ****** ********:********.** :***:* *****:********:***

MG00170.1 	SALD-------
NCU09309.1	SDFNEDDSTRV
FG06886.1 	SKLT-------
AN3932.1  	SAL--------
          	* :
```
